# Shisha Smoking Habit among Dental School Students in the United Arab Emirates: Enabling Factors and Barriers

**DOI:** 10.1155/2018/2805103

**Published:** 2018-01-31

**Authors:** Natheer H. Al-Rawi, Ahmed S. Alnuaimi, Asmaa T. Uthman

**Affiliations:** ^1^College of Dental Medicine, University of Sharjah, Sharjah, UAE; ^2^College of Medicine, University of Baghdad, Baghdad, Iraq; ^3^College of Dental Medicine, Gulf Medical University, Ajman, UAE

## Abstract

**Objectives:**

The objective of the present study was to assess shisha smoking among dental school students in Sharjah, United Arab Emirates (UAE). In addition, the role of suggested barriers and enabling factors in shisha smoking was also evaluated.

**Methods:**

A cross-sectional questionnaire-based survey was conducted at the College of Dental Medicine, University of Sharjah, between February and May 2016. The questions were adapted from previously published water pipe smoking studies. The collected data were analyzed to identify the relationship between shisha smoking and sociodemographic characteristics. Relevant questions were further categorized as enabling factors and barriers for shisha smoking.

**Results:**

Three enabling questionnaire items related to social environment were significantly associated with an increased risk of being a current smoker. The most powerful is peer pressure (“friends smoke shisha”), which increased the odds ratio of shisha smoking 11.3 times, followed by smoker sibling with increase in odd ratio by 4.52 times, then the belief of social acceptance with increase in odd ratio by 4.31 times.

**Conclusion:**

Shisha smoking is a serious problem among university students. Any intervention program in the university curricula should consider teaching students that shisha is no less risky than cigarettes and is addictive.

## 1. Introduction

Tobacco smoking is one of the ten leading health indicators proposed by the WHO Healthy People 2020 objectives [1]. According to the UN organization, by 2020, the current smoking pattern will cause about 10 million deaths every year [2]. It is estimated that death rate due to smoking will reach 8.3 million per year by the year 2030, where 70% of these deaths will occur in developing countries [3].

Tobacco consumption trend has been found constant over the past 3 decades in the Eastern Mediterranean region, especially in Iran; however, water pipe smoking was increasing in females and young people and decreasing in older people [4].

Shisha is another method of smoking that is attracting new younger customers, especially in the Middle East region. Worldwide, more than 100 million people are reported to smoke shisha [5]. The typical smoking session lasts between 45 and 50 minutes and may last for several hours [6]. Young people are attracted to shisha as the experience comes in a variety of flavors and is popular as a group activity. Many shisha users are under the misconception that shisha is less harmful than cigarettes. The reason for the drastic increase in shisha use is the false assumption that it is environmentally friendly and less harmful than cigarettes. Nevertheless, nicotine intake per shisha session has been reported as the equivalent of more than one pack of cigarettes [7]. Therefore, the type and magnitude of health adverse effects associated with shisha are prone to be different from those of cigarette smoking. Hadidi and Mohammed [7] stated that “smoking one head of flavored (Ma'assel) tobacco (204 mg/pack), containing an average of one-third of the nicotine present in 20 cigarettes, resulted in a 20% higher plasma nicotine level.”

Shisha smoking has been associated with a variety of diseases, like infertility [8], decreased respiratory function [9], infectious disease [10], and esophageal cancer [10]. The increased popularity of shisha could be attributed to its wide availability, low cost, and sociability. About 50% of university students report having used shisha, and about 25% of men currently smoke it [11]. Mohamed et al. in their study reported that around 9% of 5066 medical students in Cairo smoked both shisha and cigarettes. The prevalence of smoking was observed to be increasing from the first year through the final year despite an awareness of the hazards [12]. In Kuwait, more than 57% of men and 69% of women had used a water pipe at least once in their life [13]. In Lebanon, weekly use of water pipe was reported in 30.6% of male students and 23.4% of female students at Beirut Universities [14]. Medical student use is an example of the increased popularity of shisha smoking among educated peoples.

Among Iranian university students, 11.5% of females and 28.7% of males have been reported to smoke water pipe, compared to 2.5% of females and 18.3% of males who smoke cigarettes [15]. The sense of relaxing and energizing and a part of their culture are the main reasons behind water pipe smoking.

A systematic review of the prevalence of water pipe smoking from 38 studies found rates of current water pipe smoking in university students to be 15–28% in EMR countries, 33% in the South Asia region, 10% in the Americas, and 8% in Europe [16]. Most water pipe smokers in Ghafouri et al. study cited that social and fun aspects are the primary motivators for their continuing use of water pipe [17].

A few articles have been published about shisha or water pipe smoking among university dental students [11, 18, 19]. Questionnaire-based study among Iranian dental students revealed that 23% are current cigarette, pipe, or water pipe users [20].

However, the authors are unaware of published studies assessing shisha smoking among students of dentistry in the UAE. The aims of the present study were to estimate the prevalence rate of shisha smoking (current and ever) among students categorized by grade and sex, the nicotine dependence among current shisha smokers, and the association between the suggested barriers and enabling factors, and the shisha smoking habit was also studied.

## 2. Methods

A cross-sectional survey was conducted on students of the College of Dental Medicine at the University of Sharjah, UAE, between February and May 2016. All students from the dentistry baccalaureate program were included in the study. Students were informed about the aim of the study before the survey questionnaire was distributed.

The survey instrument was developed from a literature review and questions adapted from previously published water pipe smoking studies [19, 21].

Informed consent was obtained from each participant, and the study was approved by the research and ethics committee of the University of Sharjah. The survey questionnaire is categorized into five sections: The first section was about sociodemographic data (such as sex, age, marital status, family education, and nationality). The second section was information about the social environment like whether the student's parents, siblings, and friends smoked shisha. The third section of the questionnaire was about knowledge and awareness of shisha smoking. The fourth section consisted of questions about psychological factors involved in shisha smoking, such as stress, family and friends, and financial problems. The last section of the questionnaire addressed the personal smoking behavior of those who smoked shisha. The Modified Fagerström Test for Nicotine Dependence (MFTND) questionnaire with 5-item responses was used in this study to determine the level of dependence among the shisha smokers. The MFTND total score was calculated as the sum of the individual items, with a score of 5 or more indicating significant dependences and a score of 4 or less indicating low to moderate dependence.

The completed questionnaire was then collected and coded for data entry. Data were analyzed using IBM SPSS version 23 (IBM statistical package of social sciences program). The odds ratio was used to assess the strength of association between an independent categorical variable and an outcome dichotomous one.

## 3. Results

Out of 410 questionnaires distributed among all dental students (82 males and 328 females), only 397 students answered the questionnaire with a response rate of about 96.8%. Women constituted the highest proportion (83.1%) of the respondent students. Most of the students in the study sample were unmarried (92.7%), non-Emirati (64.6%), and had highly educated parents and high income (Table 1). The majority of participants (83.4%) mentioned that shisha was more harmful than cigarettes and that shisha smoking was addictive (76.7%), but only about one-third (37.6%) agreed that shisha was more addictive than cigarettes (Table 2). More than two-thirds of the participants acknowledged that women were more comfortable smoking shisha than cigarettes (71.3%). Most participants also acknowledged that friends could influence the shisha smoking habit (87.8%) (Table 3). Stress was found to be the most important factor influencing the start of shisha smoking (72.3%), followed by family problems (55.9%) (Table 4).

The prevalence rate of current shisha smoking among women was 8.2%, which is significantly lower than that of men (43.3%). In both sexes, the prevalence was lowest for third- and fourth-year students and varied significantly among the 3 grade categories. Among women, the prevalence was highest for the fifth and sixth years (17.9%), while among men, the prevalence was high for both the first and second and the fifth and sixth years (Table 5).

The prevalence of ever smoking shisha was much higher, being double in men compared with their current habit and triple in women. The prevalence rate of ever smoking shisha was significantly higher in men (82.1%) than in women (27.9%). Among men, the prevalence was slightly but not significantly higher among the lower grades (93.1% in the 1st to 2nd grade) and was lowest in the highest grades (72.2% in the 5th to 6th grades) (Table 6).

Of the 56 current shisha smokers, 48 completed the Modified Fagerström Nicotine Test for Dependence questionnaire (less than 20% nonresponse rate) (Table 7). A significant level of dependence was observed, which was significantly higher among men (15.4%) than among women (4.5%) (Table 8).

Some knowledge items were perceived as barriers, since they referred to the detrimental health consequences of shisha smoking and the toxic chemicals in the product. Other knowledge items were classified as enabling factors, since they encouraged shisha smoking behavior. The role of these barriers and enabling factors was tested for association with the current shisha smoking habit. This test was categorized by sex, since the perception of these knowledge items was different between the sexes.

All the 11 barrier knowledge items were more frequent among female nonsmokers than current smokers. However, only the first 2 items “shisha is more addictive than cigarettes” and “shisha is addictive” were positively identified and agreed upon by the interviewees as significantly reducing the risk of being a current shisha smoker by 5.85 and 2.38 times, respectively (Table 9). In men, 10 barriers were more frequent among nonsmokers than current shisha smokers. However, only 1 item “shisha contains nicotine” when positively identified significantly reduced the risk of being a current shisha smoker by 7.02 times. Only 3 enabling factors (items 1, 2, and 4) clearly increased the risk of being a current shisha smoker in men. These were “friends can influence shisha smoking,” “shisha is more socially acceptable compared to cigarettes,” and “people smoking shisha look cool” all of which significantly increased the risk of being current smokers by 11.08, 5.06, and 3.6, respectively (Table 10).

Among women, only 10 enabling factors clearly increased the risk of being current shisha smokers. The most powerful of these factors was item number (1) “friends smoke shisha,” which significantly increased the risk of being a current smoker by 11.32 times.

The next 4 items (items 2, 3, 5, and 6) increased the risk of current shisha smoking by more than four times. These positive knowledge items included: “smoker sibling, father, and mother,” believing that “friends can influence smoking,” and feeling that “shisha smoking is more socially acceptable than cigarettes.” In addition, agreeing that “girls are more comfortable smoking shisha compared to cigarettes” and feeling that “people smoking shisha look cool” significantly increased the risk of current shisha smoking as an outcome by more than three times. The remaining 2 knowledge items (9 and 10) “shisha smoking is our cultural heritage” and “people who smoke shisha have more friends” marginally increased the risk by between 56% and 63%. For women, the remaining 10 items originally classified as enabling factors not only did not increase the risk of current shisha smoking but also were inversely associated with this habit. These factors included stressful situations as a possible cause of smoking, including the life burden in university, the problems with friends, couple problems, and financial problems, “stress leads to shisha smoking.” Advertising about shisha was not perceived by women as an enabling factor for shisha smoking (Table 9).

Tables 11 and 12 summarize the participants' smoking habits and personal smoking behavior.

## 4. Discussion

In the present study, men with a current shisha smoking habit comprised 43.3% of the male sample, which was nearly the same percentage reported among medical students in central Saudi Arabia (44.1%) [22]. The prevalence rate was the highest in senior year students. This high prevalence could be attributed to a common belief that shisha smoking is less harmful than cigarette smoking [23]. However, in the present study, most of the male participants (86.8% nonsmokers and 79.3% current smokers) acknowledged that shisha was more harmful than cigarettes. The increasing prevalence in shisha smoking among university dental students despite the high perception of its harmful effects and the increasing trend in shisha smoking is due to other factors, including peer pressure. Akers et al. [24] have stated that “the single most direct influence on smoking among young people is how many of their 5 best friends smoke.” Initial cigarette workout occurs in the presence of other adolescents who are smoking [25, 26]. In the present study, 86.1% of surveyed nonsmokers and 96.3% of current shisha smokers supported this concept. The number of students who had ever smoked shisha was double among men and triple among women when compared with current shisha smokers (43.3% versus 82.1% for males and 8.2 versus 27.9% for females), as seen in Tables 5 and 6. More than 55.3% of nonsmokers and around 86.2% of male current smokers in the present study believed that shisha was more socially acceptable than cigarettes. Similar findings also showed that smokers do not see themselves as addicts and feel they can quit whenever they wish [27, 28]. Worldwide, more than 100 million people reported smoking shisha [4]. Young people are attracted to the water pipe as it comes in a variety of flavors and is popular as a group activity. For many years, the water pipe was considered less harmful than cigarettes; even worse many users did not consider it as a form of tobacco smoking. The use of the water pipe for smoking tobacco is a tradition in the Middle East region that goes back centuries. Since the early 1990s, however, its use has surged among new groups such as women, teenagers, and wealthy people. Shisha smoking is usually between 1 and 4 sessions/day, but with much more intense exposure per session when compared to cigarettes. Public health education and better informing health practitioners about the facts of water pipe tobacco exposure might correct the false concept of “safe smoking.” In the present study, the overall prevalence of shisha smoking was 14.1% among dental students. A lower prevalence rate (5.6% and 7.3%) was reported among other nonmedical students at the University of Sharjah, UAE [1], and university students in Riyadh, Saudi Arabia [29].

A study conducted in the United Kingdom on 181 dental students showed that more than 90% of dental students had moderate or good knowledge and more than 80% had an appropriate attitude towards smoking [30]. However, appropriate knowledge and attitude alone cannot result in a proper behavior, and other confounding factors like socioeconomic and family issues [31, 32] should also be taken into account [3].

The Modified Fagerström Nicotine Test of Dependence (MFNTD) questionnaire with 5-item responses was used in the present study to determine the level of dependence (Table 7). The total score was calculated as the sum of the individual items; a significant level of dependence was observed in 15.4% of men with a current shisha habit (Table 8). Ghafouri et al. [17] found that 17.2% of their studied sample felt that shisha smoking was addictive.

Stress was found to be one of the enabling factors that leads to shisha smoking (22/29 men and 17/27 women) among current shisha smokers as shown in Tables 9 and 10. The high perception of the harmful effect of shisha smoking among participants does not prevent them from practicing this habit. This may be contributing to the widespread of this type and other types of tobacco use. Similarly, a study done at the American University of Beirut [32] reported that most students are aware about the effects of smoking on health but lacked detailed information about how these effects happened. These discrepancies reflect the failure of university curricula in this region to teach students about the harmful properties of tobacco effectively.

In addressing the concept of barriers, acknowledging the addictive nature of shisha and the fact that it is more addictive than cigarettes was found to reduce the risk of being a current shisha smoker among women. These barriers did not affect the male habit, in fact their knowledge about the nicotine content of shisha increased the risk of the habit.

Among the factors that promote the habit of smoking shisha, both sexes agreed on the positive peer effect. However, current female shisha smokers were more likely to have a friend with the shisha smoking habit. In addition, both sexes were significantly affected by the positive social image of shisha. They used the word “cool” to describe the habit. In addition, they thought that shisha smoking was more acceptable than cigarettes. Socially, women are more comfortable with shisha smoking than cigarette smoking. Finally, the role of family members (father, mother, and siblings) was an important promoter for women.

If dental students are to become advocates for reducing smoking, the dental schools' curricula should stress more about the awareness of tobacco-related diseases. The positive social image of shisha for both sexes and the role model of family members in fostering this habit among women should be addressed to prevent its spread. The increasing number of women smoking water pipes may become an epidemic unless robust health education programs are designed to combat water pipe smoking, especially in these age groups.

## 5. Conclusion

Water pipe smoking among university students is of growing concern. Acknowledging the addictive nature of shisha and the fact that it is more addictive than cigarettes is an important barrier for the habit among women users. Among the factors that promote the habit in both sexes were the positive peer effect and the positive social image of shisha. The influential role model of other family members smoking shisha was an important promoter only for women. An intervention program to prevent this health problem must teach the public that water pipes are no less risky than cigarettes. Water pipe smoking should be bound by the same regulations and laws as cigarette smoking, including banning it in public places.

Furthermore, teachers and faculty at schools and universities should be engaged to include tobacco and water pipe prevention programs in their curriculum.

## Figures and Tables

**Table 1:** Sociodemographic characteristics of the study participants (*n* = 397).

Variables	Categories	Number	Percentage (%)
Sex	Females	330	83.1
	Males	67	16.9
	Total	397	100.0

Grades	First and second	160	40.3
	Third and fourth	141	35.5
	Fifth and sixth	96	24.1
	Total	397	100.0

Being married	Single	368	92.7
	Married	29	7.3
	Total	397	100.0

Father education	Illiterate/read and write	18	4.6
	Elementary/intermediate school	19	4.8
	Secondary school	60	15.11
	University and higher education	296	75.55
	Not answered	4	0.01
	Total	397	100.0

Mother education	Illiterate/read and write	15	3.8
	Elementary/intermediate school	25	6.3
	Secondary school	86	21.66
	University and higher education	268	67.5
	Not answered	3	0.007
	Total	397	100.0

Nationality	UAE citizen	141	35.5
	Non-UAE citizen	256	64.5
	Total	397	100.0

**Table 2:** Knowledge and awareness about shisha smoking.

Variables	Number	Percentage (%)
Shisha contains nicotine	349	87.9
Shisha is addictive	304	76.6
Shisha is more addictive than cigarettes	150	37.6
Shisha is more harmful compared with cigarettes	331	83.4
Shisha smoking leads to lung cancer	367	92.4
Shisha smoking leads to cardiovascular diseases	369	92.9
Shisha smoking leads to dental problems	375	94.5
Shisha smoking reduces weight	183	46.1
Shisha contains carbon monoxide	363	91.4
Water in shisha filters toxins	139	35
Shisha is harmful to health	382	96.2
Shisha contains tar	284	71.5

**Table 3:** Social environment characteristics of the study participants.

Variables	Number	Percentage (%)
Father smokes shisha	48	12.1
Mother smokes shisha	23	5.8
Sibling smokes shisha	137	34.5
Friends can influence smoking	348	87.7
Parents influenced decision to start shisha smoking	192	48.4
Advertisement about shisha can influence smoking	276	69.5
Girls are more comfortable in smoking shisha compared to cigarette	283	71.3
People smoking shisha look cool	58	14.6
Shisha smoking is our cultural heritage	108	27.2
People smoking shisha have more friends	70	17.6

**Table 4:** Psychological factors involved in shisha smoking.

Variables	Number	Percentage (%)
University life burden leads to shisha smoking	186	46.9
Stress influences one to start shisha smoking	287	72.3
Problems with friends lead to shisha smoking	137	34.6
Husband-wife problems lead to shisha smoking	179	45.1
Family problems lead to shisha smoking	222	55.9
Financial problems lead to shisha smoking	143	36.0

**Table 5:** Prevalence rate of current shisha smoking stratified by grade and sex.

	Total	Current shisha smoker	%	95% confidence interval	*P*
	*N*	*N*			
*Female*					<0.001
First and second grade	131	10	7.6	(3.1 to 12.2)	
Third and fourth grade	121	3	2.5	(−0.3 to 5.2)	
Fifth and sixth grade	78	14	17.9	(9.4 to 26.5)	
Total female	330	27	8.2	(5.2 to 11.1)	
*Male*					0.04
First and second grade	29	16	55.2	(37.1 to 73.3)	
Third and fourth grade	20	4	20.0	(2.5 to 37.5)	
Fifth and sixth grade	18	9	50.0	(26.9 to 73.1)	
Total male	67	29	43.3	(31.4 to 55.1)	

*P* (chi-square) for male-female differences is <0.00.

**Table 6:** Prevalence rate of ever smoked shisha stratified by grade and sex.

	Total	Ever smoked shisha	%	95% confidence interval	*P*
	*N*	*N*			
*Female*					0.002
First and second grade	131	40	30.5	(22.6 to 38.4)	
Third and fourth grade	121	21	17.4	(10.6 to 24.1)	
Fifth and sixth grade	78	31	39.7	(28.9 to 50.6)	
Total female	330	92	27.9	(23 to 32.7)	
*Male*					0.12 (NS)
First and second grade	29	27	93.1	(83.9 to 102.3)	
Third and fourth grade	20	15	75.0	(56 to 94)	
Fifth and sixth grade	18	13	72.2	(51.5 to 92.9)	
Total male	67	55	82.1	(72.9 to 91.3)	

*P* (chi-square) for male-female differences is <0.00.

**Table 7:** The Modified Fagerström Nicotine Test for Dependence scale.

(1) How soon after you wake up do you smoke your first shisha:	
(i) Within 5 minutes	3
(ii) 6–30 minutes	2
(iii) 31–60 minutes	1
(iv) After 60 minutes	0
(2) Which shisha smoking would you hate most to give up?	
(i) The first one in the morning	1
(ii) Any other	0
(3) How many Hager/week?	
(i) 1 per week	0
(ii) 2–4 per week	1
(iii) More than 5 per week	2
(4) Do you smoke more frequently during the first hours after waking than during the rest of the day?	
(i) Yes	1
(ii) No	0
(5) Do you smoke if you are so ill that you are in bed most of the day?	
(i) Yes	1
(ii) No	0

**Table 8:** Nicotine dependence level among current shisha smokers by sex.

	Fagerström's Nicotine Tolerance score categories					
	Low level of dependence		Moderate level of dependence		Total	
	*N*	%	*N*	%	*N*	%
*Sex*						
Female	21	95.5	1	4.5	22	100
Male	22	84.6	4	15.4	26	100
*P* (Fisher's exact) = 0.35 (NS)						

**Table 9:** The strength of association between selected barriers and enabling factors with current shisha smoking habits among females.

Sex = female	Current shisha smoking habit				OR	Inverse OR	95% confidence interval OR	*P*
	Nonsmokers (*N* for total surveyed = 303)		Current smokers (*N* for total surveyed = 27)					
	*N*	%	*N*	%				
*Barriers*								
(1) Shisha is more addictive than cigarettes	128	42.2	3	11.1	0.17	5.85	(0.05–0.58)	**0.005**
(2) Shisha is addictive	243	80.2	17	63	0.42	2.38	(0.18–0.96)	**0.041**
(3) Shisha smoking leads to lung cancer	286	94.4	24	88.9	0.48	2.10	(0.13–1.74)	0.261 (NS)
(4) Shisha smoking leads to dental problems	290	95.7	25	92.6	0.56	1.78	(0.12–2.62)	0.462 (NS)
(5) Shisha smoking reduces weight	150	49.5	10	37	0.60	1.67	(0.27–1.35)	0.218 (NS)
(6) Shisha is more harmful compared with cigarettes	254	83.8	21	77.8	0.68	1.48	(0.26–1.76)	0.421 (NS)
(7) Shisha contains carbon monoxide	277	91.4	24	88.9	0.75	1.33	(0.21–2.66)	0.657 (NS)
(8) Shisha is harmful to health	294	97	26	96.3	0.80	1.26	(0.1–6.53)	0.832 (NS)
(9) Shisha contains nicotine	273	90.1	24	88.9	0.88	1.14	(0.25–3.09)	0.841 (NS)
(10) Shisha smoking leads to cardiovascular diseases	282	93.1	25	92.6	0.93	1.07	(0.21–4.2)	0.926 (NS)
(11) Shisha contains tar	217	71.6	21	77.8	1.39	—	(0.54–3.55)	0.496 (NS)
*Enabling factors*								
(1) Friends smoke shisha	159	52.5	25	92.6	11.32	—	(2.63–48.65)	**0.001**
(2) Smoker sibling	93	30.7	18	66.7	4.52	—	(1.96–10.43)	**<0.001**
(3) Shisha smoking is more socially acceptable compared to cigarettes	153	50.5	22	81.5	4.31	—	(1.59–11.7)	**0.004**
(4) Friends can influence smoking	261	86.1	26	96.3	4.18	—	(0.55–31.7)	0.166 (NS)
(5) Father smokes shisha	33	10.9	9	33.3	4.09	—	(1.7–9.84)	**0.002**
(6) Mother smokes shisha	16	5.3	5	18.5	4.08	—	(1.37–12.17)	**0.012**
(7) Girls are more comfortable in smoking shisha compared to cigarettes	204	67.3	24	88.9	3.88	—	(1.14–13.22)	**0.03**
(8) People smoking shisha look cool	31	10.2	7	25.9	3.07	—	(1.2–7.84)	**0.019**
(9) Shisha smoking is our cultural heritage	71	23.4	9	33.3	1.63	—	(0.7–3.8)	0.254 (NS)
(10) People smoking shisha have more friends	47	15.5	6	22.2	1.56	—	(0.6–4.06)	0.366 (NS)
(11) University life burden leads to shisha smoking	143	47.2	12	44.4	0.90	1.12	(0.41–1.98)	0.784 (NS)
(12) Water in shisha filters toxins	105	34.7	7	25.9	0.66	1.52	(0.27–1.61)	0.362 (NS)
(13) Stress influences one to start shisha smoking	224	73.9	17	63	0.60	1.67	(0.26–1.36)	0.223 (NS)
(14) Problems with friends lead to shisha smoking	113	37.3	6	22.2	0.48	2.08	(0.19–1.23)	0.125 (NS)
(15) Shisha smoker parents influenced decision of children to start shisha smoking	181	59.7	11	40.7	0.46	2.16	(0.21–1.03)	0.06 (NS)
(16) Parents influenced decision of children to start shisha smoking	160	52.8	9	33.3	0.45	2.24	(0.19–1.03)	0.058 (NS)
(17) Husband-wife or boyfriend-girlfriend problems lead to shisha smoking	149	49.2	8	29.6	0.44	2.30	(0.18–1.03)	0.057 (NS)
(18) Family problem leads to shisha smoking	183	60.4	9	33.3	0.33	3.05	(0.14–0.75)	**0.009**
(19) Financial problems lead to shisha smoking	122	40.3	4	14.8	0.26	3.88	(0.09–0.76)	**0.015**
(20) Advertisement about shisha can influence smoking	220	72.6	9	33.3	0.19	5.30	(0.08–0.44)	**<0.001**

**Table 10:** The strength of association between selected barriers and enabling factors with current shisha smoking habits among males.

Sex = male	Current shisha smoking habit				OR	Inverse OR	95% confidence interval OR	*P*
	Nonsmokers (*N* for total surveyed = 38)		Current smoker (*N* for total surveyed = 29)					
	*N*	%	*N*	%				
*Barriers*								
(1) Shisha is more addictive than cigarettes	36	94.7	26	89.7	0.48	2.08	(0.08–3.09)	0.441 (NS)
(2) Shisha is addictive	13	34.2	6	20.7	0.50	1.99	(0.16–1.54)	0.228 (NS)
(3) Shisha smoking leads to lung cancer	33	86.8	23	79.3	0.58	1.72	(0.16–2.13)	0.413 (NS)
(4) Shisha smoking leads to dental problems	26	68.4	18	62.1	0.76	1.32	(0.27–2.08)	0.588 (NS)
(5) Shisha smoking reduces weight	25	65.8	27	93.1	7.02	—	(1.44–34.29)	**0.016**
(6) Shisha is more harmful compared with cigarettes	34	89.5	28	96.6	3.29	—	(0.35–31.19)	0.299 (NS)
(7) Shisha contains carbon monoxide	32	84.2	25	86.2	1.17	—	(0.3–4.61)	0.82 (NS)
(8) Shisha is harmful to health	34	89.5	26	89.7	1.02	—	(0.21–4.96)	0.981 (NS)
(9) Shisha contains nicotine	13	34.2	10	34.5	1.01	—	(0.37–2.8)	0.981 (NS)
(10) Shisha smoking leads to cardiovascular diseases	34	89.5	28	96.6	3.29	—	(0.35–31.19)	0.299 (NS)
*Enabling factors*								
(1) Friends can influence shisha smoking	32	84.2	29	100	11.80	—	(1.37–101.5)	**0.025**
(2) Shisha smoking is more socially acceptable compared to cigarettes	21	55.3	25	86.2	5.06	—	(1.47–17.4)	**0.01**
(3) Girls are more comfortable in smoking shisha compared to cigarettes	28	73.7	27	93.1	4.82	—	(0.97–24.05)	0.055 (NS)
(4) People smoking shisha look cool	7	18.4	13	44.8	3.60	—	(1.2–10.81)	**0.022**
(5) Friends smoke shisha	31	81.6	27	93.1	3.05	—	(0.58–15.94)	0.187 (NS)
(6) Smoker sibling	12	31.6	14	48.3	2.02	—	(0.74–5.5)	0.167 (NS)
(7) Husband-wife or boyfriend-girlfriend problems lead to shisha smoking	10	26.3	12	41.4	1.98	—	(0.7–5.55)	0.196 (NS)
(8) Stress influences one to start shisha smoking	24	63.2	22	75.9	1.83	—	(0.63–5.38)	0.27 (NS)
(9) Water in shisha filters toxins	13	34.2	14	48.3	1.79	—	(0.67–4.83)	0.247 (NS)
(10) Financial problems lead to shisha smoking	8	21.1	9	31	1.69	—	(0.56–5.11)	0.355 (NS)
(11) Mother smokes shisha	1	2.6	1	3.4	1.32	—	(0.08–22.06)	0.846 (NS)
(12) Shisha smoker parents influenced decision of children to start shisha smoking	13	34.2	11	37.9	1.18	—	(0.43–3.21)	0.753 (NS)
(13) University life burden leads to shisha smoking	17	44.7	14	48.3	1.15	—	(0.44–3.04)	0.774 (NS)
(14) Problems with friends lead to shisha smoking	10	26.3	8	27.6	1.07	—	(0.36–3.17)	0.907 (NS)
(15) Family problem leads to shisha smoking	17	44.7	13	44.8	1.00	—	(0.38–2.65)	0.994 (NS)
(16) People smoking shisha have more friends	10	26.3	7	24.1	0.89	1.12	(0.29–2.72)	0.839 (NS)
(17) Advertisement about shisha can influence smoking	28	73.7	19	65.5	0.68	1.47	(0.24–1.94)	0.47 (NS)
(18) Father smokes shisha	4	10.5	2	6.9	0.63	1.59	(0.11–3.7)	0.609 (NS)
(19) Parents influenced decision of children to start shisha smoking	15	39.5	8	27.6	0.58	1.71	(0.21–1.66)	0.312 (NS)
(20) Shisha smoking is our cultural heritage	18	47.4	10	34.5	0.58	1.71	(0.22–1.58)	0.291 (NS)

**Table 11:** Participant's smoking habits and dependence (current shisha smokers).

Variables	Number	Percentage (%)
Current shisha smoking	56	14.1
Share shisha	87	42.8
Smoke shisha at café	105	51.7
Smoke shisha with friends	96	42.2
Smoke cigarettes	41	20.1
Mixed smoker	43	21.1
How often do you smoke shisha?		
(i) 1/week	73	66.3
(ii) 2–4/week	21	19
(iii) More than 5/week	16	14.5
Number of Hager/day		
(i) One	28	77.7
(ii) More than 1	8	22.2
Number of Hager/week		
(i) One	23	53.5
(ii) 2–4	12	27.9
(iii) More than 5	8	18.6
Minutes to first shisha of the day		
(i) Less than hour	46	43.8
(ii) More than hour	59	56.2
How soon do you smoke after you wake up		
(i) Less than hour	46	43.8
(ii) More than hour	59	46.2
Which shisha smoking would you hate to give up		
(i) First one in the morning	18	18.9
(ii) Any other in the afternoon or evening	77	81.1
Do you find difficulty to refrain?		
(i) Yes	29	25.2
(ii) No	86	74.8
Do you smoke when ill		
(i) Yes	28	23.7
(ii) No	90	76.3
Do you inhale smoking		
(i) Yes	44	38.2
(ii) No	71	61.8
Do you want to quit?		
(i) Yes	51	44.7
(ii) No	63	55.3
Did you try to quit shisha smoking?		
(i) Yes	43	38
(ii) No	70	6262

**Table 12:** Personal smoking behavior.

		*N*	%
Current shisha smoking habit	Negative	341	85.9
	Current shisha smoker	56	14.1
	Total	397	100.0

Ever smoked shisha	Negative	250	63.0
	Positive	147	37.0
	Total	397	100.0

Shisha smoking habit	Negative	250	63.0
	Ex-shisha smoker	91	22.9
	Current shisha smoker	56	14.1
	Total	397	100.0

Shares shisha with others	Negative	14	25.0
	Positive	42	75.0
	Total	56	100.0

Smoke shisha usually at café	Negative	2	3.6
	Positive	54	96.4
	Total	56	100.0

Smoking shisha with friends/family	Negative	6	10.7
	Positive	50	89.3
	Total	56	100.0

Smokes shisha occasionally	Negative	10	17.9
	Positive	46	82.1
	Total	56	100.0

Smoke cigarettes	Negative	41	73.2
	Positive	15	26.8
	Total	56	100.0

Mixed smoker	Negative	38	67.9
	Positive	18	32.1
	Total	56	100.0

Frequency of smoking shisha	≤1/week	38	67.9
	2–4 sessions/week	11	19.6
	5 ± week	7	12.5
	Total	56	100.0

Dose of smoking per week (Hager/week)-categories	Only one	34	66.7
	2–4	10	19.6
	5–9	6	11.8
	10+	1	2.0
	Total	51	100.0

Duration of first shisha of the day	0	32	61.5
	1	20	38.5
	Total	52	100.0

Do you smoke more during the first 2 hours of the day than during the rest of the day	Negative	50	92.6
	Positive	4	7.4
	Total	54	100.0

How soon after you wake up do you smoke your first shisha	In the afternoon or evening	49	94.2
	>30 min but before noon	2	3.8
	<30 min	1	1.9
	Total	52	100.0

Which shisha smoking would you hate to give up	Any other in the afternoon or in the evening	4	8.0
	First one in the morning	46	92.0
	Total	50	100.0

Difficult to refrain from shisha smoking in places where it is forbidden	Negative	44	81.5
	Positive	10	18.5
	Total	54	100.0
Smoking when ill	Negative	43	78.2
	Positive	12	21.8
	Total	55	100.0

Inhalation of smoking	Negative	35	64.8
	Positive	19	35.2
	Total	54	100.0

Want to quit shisha smoking	Negative	30	54.5
	Positive	25	45.5
	Total	55	100.0

Attempt to quit shisha smoking	Negative	36	65.5
	Positive	19	34.5
	Total	55	100.0

Modified Fagerström Test for Nicotine Dependence score categories	Low level of dependence	43	89.6
	Moderate level of dependence	5	10.4
	Total	48	100.0
